# Protective Effects of L-Malate against Myocardial Ischemia/Reperfusion Injury in Rats

**DOI:** 10.1155/2016/3803657

**Published:** 2016-01-31

**Authors:** Shiao Ding, Yang Yang, Ju Mei

**Affiliations:** Department of Cardiothoracic Surgery, Xin Hua Hospital, Shanghai Jiao Tong University School of Medicine, Shanghai 200092, China

## Abstract

*Objective*. To investigate the protective effects of L-malate against myocardial ischemia/reperfusion (I/R) injury in rats.* Methods*. Male Sprague-Dawley rats were randomly assigned to the following groups: sham (sham), an ischemia/reperfusion (I/R) model group (model), an DMF pretreated group (DMF), and 5 L-malate pretreated groups (15, 60, 120, 240, or 480 mg/kg, gavage) before inducing myocardial ischemia. Plasma LDH, cTn-I, TNF-*α*, hs-CRP, SOD, and GSH-PX were measured 3 h later I/R. Areas of myocardial infarction were measured; hemodynamic parameters during I/R were recorded. Hearts were harvested and Western blot was used to quantify Nrf2, Keap1, HO-1, and NQO-1 expression in the myocardium.* Results*. L-malate significantly reduced LDH and cTn-I release, reduced myocardial infarct size, inhibited expression of inflammatory cytokines, and partially preserved heart function, as well as increasing antioxidant activity after myocardial I/R injury. Western blot confirmed that L-malate reduced Kelch-like ECH-associated protein 1 in ischemic myocardial tissue, upregulated expression of Nrf2 and Nrf2 nuclear translocation, and increased expression of heme oxygenase-1 and NAD(P)H:quinone oxidoreductase 1, which are major targets of Nrf2.* Conclusions*. L-malate may protect against myocardial I/R injury in rats and this may be associated with activation of the Nrf2/Keap1 antioxidant pathway.

## 1. Introduction

Ischemic heart disease contributes to high health care costs and mortality worldwide [1]. Therapeutic strategies to restore cardiac blood flow to the ischemic myocardium such as thrombolysis, primary angioplasty, and cardiac surgery under cardiopulmonary bypass (CPB) are commonly used. During cardiac surgery under CPB, ischemic cardiac arrest is initiated and perfusion is restored. However, data show that the reperfusion itself is damaging and can exacerbate necrosis, producing myocardial ischemia/reperfusion injury (MIRI) that can influence surgical treatment and postoperative long-term recovery [2–4]. The mechanism of MIRI is unclear and better treatment strategies are needed.

Recently, drugs that offer cardioprotective effects have been studied but few have made it to clinical use, perhaps due to animal model choices for studying drug effects as well as myriad side effects and limited efficacy [5–7]. Fumaric acid, a tricarboxylic acid cycle small molecule metabolite, may offer myocardial protection via activation of the Nrf2 antioxidant pathway [8]. Thus, we speculate that similar compounds may also offer myocardial protection. A structural analogue of fumaric acid, L-malate, is produced during biological metabolism of organic acids and serves as an important metabolic intermediate. In 1967, L-malic acid was recognized as a safe, nontoxic, harmless, and edible organic acid by the US Food and Drug Administration [9]. As a tricarboxylic acid cycle intermediate, L-malate can be easily absorbed into the mitochondrion through the cell membrane, and here it promotes energy production. Research suggests that some organic acids have various pharmacological effects and biological activity, including anti-inflammatory, antiplatelet aggregation, and antioxidant activity, and may reduce apoptosis [10–14]. Thus, our work with L-malate on MIRI is new and preliminary. We report here that L-malate may protect the heart from MIRI and we have postulated a possible mechanism by which it offers myocardial protection.

## 2. Materials and Methods

### 2.1. Animals

Male adult Sprague-Dawley rats (200–250 g) were purchased from Chinese Academy of Sciences Holdings Co., Ltd. (certificate number SCXK (HU) 2012-0002). Rats were housed under standard conditions and supplied with drinking water and food* ad libitum*. All animal experiments were performed in accordance with the China Academy of Chinese Medical Sciences Guide for Laboratory Animals which conforms to the Guide for the Care and Use of Laboratory Animals published by the US National Institutes of Health (NIH Publications number 85-23, revised 1996).

### 2.2. Reagents and Chemicals

An LDH assay kit was purchased from Shanghai Ailex Technology Co., Ltd. (batch number R403ACA). A superoxide dismutase (SOD) assay kit (batch number 20150126) and glutathione peroxidase (GSH-PX) assay kit (batch number 20150124) were obtained from Nanjing Jiancheng Bioengineering Institute. Rat tumor necrosis factor-*α* (TNF-*α*) (Catalog number bs-0078R), rat high-sensitivity-C-reactive protein (hs-CRP) (Catalog number bs-0078R), and cTn-I Quantikine ELISA kit (Catalog number 20150126) were obtained from Shanghai J&L Biological Technology Co., Ltd. Nitroblue tetrazolium (N-BT) (Catalog number 3069B117) was from AMRESCO Inc. (Solon, OH). Antibodies for actin (number sc-1616r), anti-Nrf2 (number sc-722), anti-HO-1 (sc-1797), and anti-NQO-1 (sc-25591) were from Santa Cruz Biotechnology Inc. (Santa Cruz, CA), and anti-Keap1 (number bs6783) was from Bio world Technology Inc. (St Louis Park, MN). L-malate (batch number J1427119) and dimethyl fumarate (batch number K1405001) were purchased from Shanghai Jingchun Biochemical Technology Co., Ltd. All chemicals used were of analytical grade.

### 2.3. Drug Pretreatment and Myocardial Ischemia/Reperfusion Protocols

L-malate studies on MIRI were performed independently with identical experimental designs. Animals were randomly assigned to 8 groups (*N* = 10): sham (sham), ischemia/reperfusion (I/R) model group (model), DMF pretreated group (DMF), and the 5 L-malate pretreated groups (15, 60, 120, 240, or 480 mg/kg, gavage), respectively, upon initiation of myocardial ischemia. Sham animals received surgery only (sham-operated), and other groups were subjected to myocardial ischemia and reperfusion 30 min later. In addition, the sham and I/R model control groups were gavaged an equal volume of L-malate vehicle. Vehicle/drugs were administered twice daily for 5 consecutive days prior to the experiment.

### 2.4. Myocardial I/R Injury

MIRI was carried out via LAD ligation for 30 min followed by 3 h reperfusion at 1 h after the last drug treatment as previously described. Rats were anesthetized with 3.5% chloral hydrate (350 mg/kg, ip, Sinopharm Chemical Reagent Co., Ltd., China). The trachea was exposed and cannulated to establish artificial respiration provided by a rodent ventilator (ALCV8S, China) with oxygen at a breath ratio of 1 : 2; a frequency of 75 breaths/min, and a tidal volume of 8.0 mL. MI was produced by exteriorizing the heart through a left thoracic incision and placing a 4-0 silk suture and placing plastic tubing at the distal one-third of the left anterior descending coronary artery. After 30 min of ischemia, the plastic tubing was cut and the myocardium was reperfused.

### 2.5. Hemodynamic Assessment

We separated the right common carotid artery and connected the RM6240 biological signal processing system to monitor heart function, including left ventricular systolic pressure (LVSP), left ventricular end-diastolic pressure (LVEDP), and first derivative (±*dp*/*dt*_max_) of left ventricular pressure in each group. To eliminate confounding factors of loading conditions of the heart which may influence cardiovascular parameters, additional rats were used to test whether L-malate alone modified LVSP, LVEDP, and ±*dp*/*dt*_max_ in normal hearts under sham-operated conditions.

### 2.6. Measurement of Myocardial Infarct Size

Myocardial infarct size was evaluated using N-BT staining as previously described [5]. Briefly, at the end of 3 h reperfusion, rats were anesthetized with 3.5% chloral hydrate and sacrificed. Hearts were quickly excised and sliced into 6 sections from the position under the ligation line (1.5 to 2 mm). Slices were weighed and immediately incubated in N-BT staining solution dissolved in phosphate buffer (pH 7.4) at 37°C for 10 min. The noninfarcted myocardia were uniformly blue, and the infarction area was not stained or appeared pale yellow. The infarcted weights and left ventricular weights were measured using an electronic balance (FA1104, Shanghai, China). The infarction percentages of the ventricle were calculated.

### 2.7. Serum Biochemistry

After 3 h of reperfusion, blood samples were collected from the right ventricle and centrifuged at 3,000 ×g for 10 min to isolate sera. Then, LDH, SOD, and GSH-PX were measured with kits according to the manufacturer's instructions. ELISA was used to quantify cTn-I, TNF-*α*, and hs-CRP.

### 2.8. Western Blot

Nrf2, Keap1, HO-1, and NQO-1 expression were measured using Western blot using myocardial samples extracted after 3 h of reperfusion. Nuclear and cytoplasmic protein were isolated (Pierce, Rockford, IL) according to the manufacturer's instructions. Then, whole heart protein was extracted from total homogenous of heart tissue. In brief, heart tissue was first ground in liquid nitrogen and lysed in ice-cold T-PER buffer containing 1% protease inhibitor cocktail (Pierce). Then homogenates were incubated on ice for 30 min before centrifugation at 12,000 ×g for 10 min at 4°C. Supernatant was transferred, aliquot, and stored at –20°C. After protein was measured using a modified Bradford assay (Bio-Rad Laboratories, Hercules, STATE), proteins were separated on SDS-PAGE and transferred to nitrocellulose membranes and probed with primary antibodies against Nrf2 (1 : 1,000), HO-1 (1 : 500), NQO1 (1 : 1,000), and Keap1 (1 : 1,000), overnight at 4°C followed by incubation with corresponding secondary antibodies at room temperature for 1 h. Blots were visualized with ECL-Plus reagent ECL Western Blotting Substrate (Pierce, Rockford, IL).

### 2.9. Statistical Analysis

All data are presented as means ± SD. Statistical analysis was performed using SPSS 16.0 and data were analyzed with ANOVA analysis followed by Student-Newman-Keuls test for multiple comparisons. In all cases, *P* < 0.05 was considered to be a statistically significant difference.

## 3. Results

### 3.1. L-Malate Improves Functional Recovery after I/R Injury

**Figure 1:**
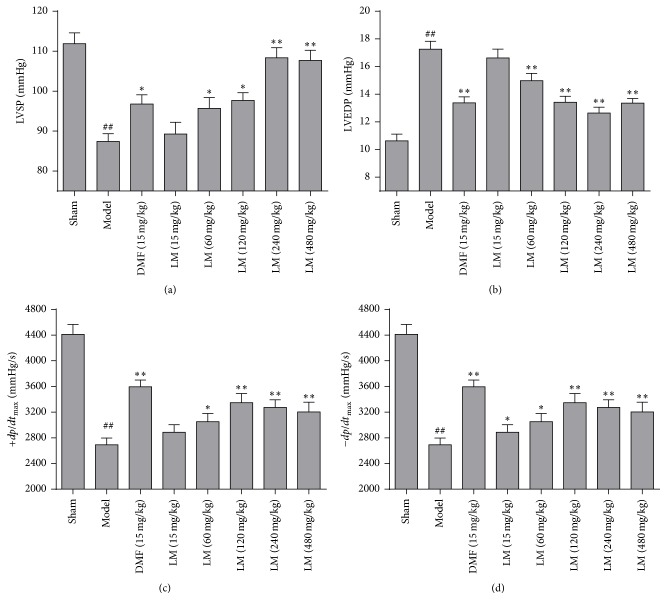
L-malate (LM) improves functional recovery after I/R injury. Data are means ± SD. ^##^ *P* < 0.01 versus sham, ^*∗∗*^ *P* < 0.01, ^*∗*^ *P* < 0.05 versus model. LVSP: left ventricular systolic pressure; LVEDP: left ventricular end-diastolic pressure; +*dp*/*dt*_max_: indices of left ventricular contraction; −*dp*/*dt*_max_: indices of left ventricular relaxation.

No significant difference in LVEDP, LVSP, and ±*dp*/*dt*_max_ was observed in all animals before MIRI (Table 1). Hemodynamic changes recorded in anesthetized animals are presented in Figure 1. After myocardial I/R injury, LVSP and ±*dp*/*dt*_max_ decreased, and LVEDP was significantly increased in controls compared to shams. These effects were partly reversed after treatment with DMF and L-malate (≥60 mg/kg) and these effects were L-malate concentration-dependent.

### 3.2. L-Malate Reduces LDH and cTn-I Release after I/R Injury

**Figure 2:**
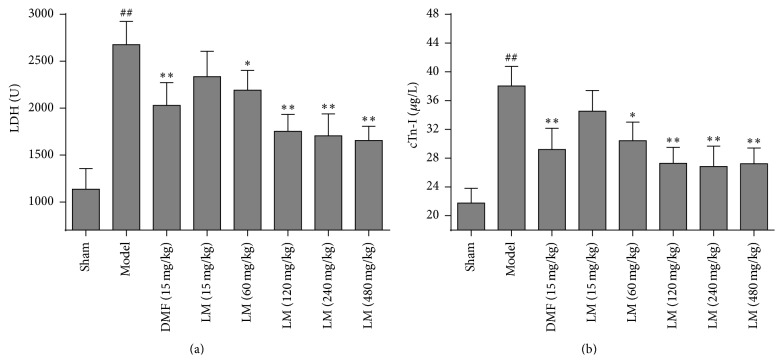
L-malate (LM) reduces myocardial damage after MIRI. (a) LDH and (b) cTn-I were assayed 3 h after reperfusion. Data are presented as means ± SD from 8 experiments. ^##^ *P* < 0.01 versus sham, ^*∗∗*^ *P* < 0.01, ^*∗*^ *P* < 0.05 versus model.

Compared with shams, LDH and cTn-I in models were significantly increased, and these were significantly different (*P* < 0.01) (Figure 2). Compared with models, DMF reduced cTn-I and LDH (*P* < 0.05 or *P* < 0.01); L-malate (120 mg and greater) reduced cTn-I and LDH as well (*P* < 0.01).

### 3.3. L-Malate Reduces Myocardial Damage after I/R Injury

**Figure 3:**
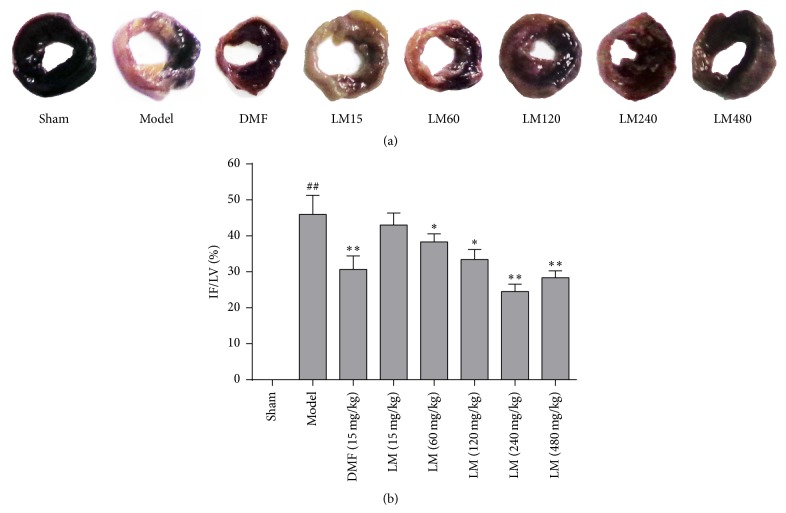
L-malate (LM) decreases infarct volume after MIRI. Representative N-BT staining of infarct size. Noninfarcted myocardial stained dark blue, and the ischemic area was white or grayish-yellow. Myocardial infarct volume was assayed 3 h after reperfusion. Data are presented as means ± SD from 8 experiments. ^##^ *P* < 0.01 versus sham, ^*∗∗*^ *P* < 0.01, ^*∗*^ *P* < 0.05 versus model.

To investigate the potential protective effects of L-malate against MIRI, we measured the myocardial infract volume.  Figure 3 shows that no MI occurred in the sham group, but MIRI was significant (*P* < 0.01) in the other groups and DMF significantly reduces the ventricular infarction compared to model (*P* < 0.01). L-malate reduces infarct volume in a dose-dependent manner at concentrations greater than 15 mg/kg, which was not different than the vehicle group (*P* > 0.05). The highest dose (240 mg/kg) of L-malate was the most effective and more protection was not observed at higher concentrations.

### 3.4. Effects of L-Malate and Inflammatory Cytokine Expression

**Figure 4:**
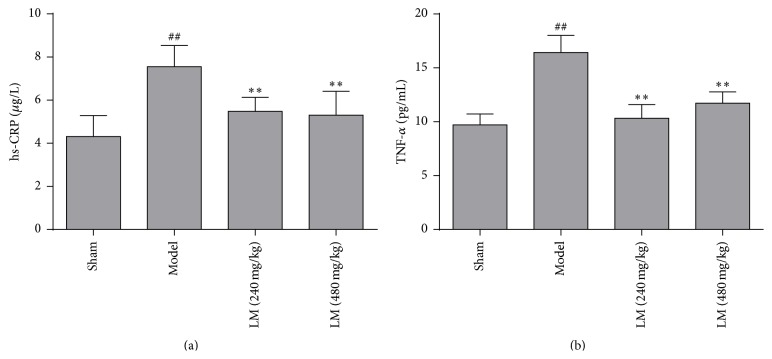
L-malate (LM) attenuates expression of inflammatory cytokines after MIRI. (a) hs-CRP and (b) TNF-*α* were assayed 3 h after MIRI. Data are presented as means ± SD from 4 experiments. ^##^ *P* < 0.01 versus sham, ^*∗∗*^ *P* < 0.01, ^*∗*^ *P* < 0.05 versus model.

To investigate potential anti-inflammatory activity of L-malate after MIRI, cytokines associated with inflammation, such as hs-CRP and TNF-*α*, were measured. Compared with sham, hs-CRP and TNF-*α* significantly were increased (*P* < 0.01) in model controls. In groups treated with L-malate (240 and 480 mg/kg) hs-CRP and TNF-*α* decreased compared to model controls, but, at higher concentrations of L-malate, TNF-*α* increased (Figure 4).

### 3.5. Effects of L-Malate on Antioxidant Activity after MIRI

**Figure 5:**
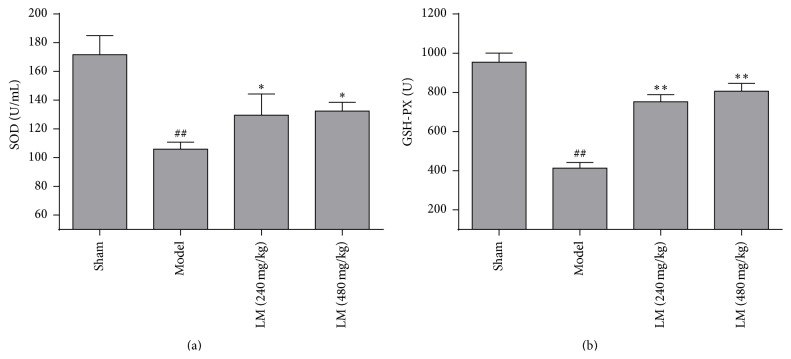
L-malate (LM) increases antioxidant enzyme activity after MIRI. (a) SOD and (b) GSH-PX were measured by ELISA 3 h after MIRI. Data are presented as means ± SD from 4 experiments. ^##^ *P* < 0.01 versus sham group, ^*∗∗*^ *P* < 0.01 versus model.

Antioxidants such as SOD and GSH-PX were increased in model controls compared to shams 3 h after MIRI, suggesting oxidative damage conferred by MIRI (Figure 5). Treatment with L-malate significantly preserved SOD and GSH-PX activity 3 h after MIRI and this was dose-dependent (*P* < 0.05 or *P* < 0.01).

### 3.6. Effects of L-Malate on Expression of Keap1, Nrf2, HO-1, NQO-1, and Nrf2 Nuclear Translocation after MIRI

**Figure 6:**
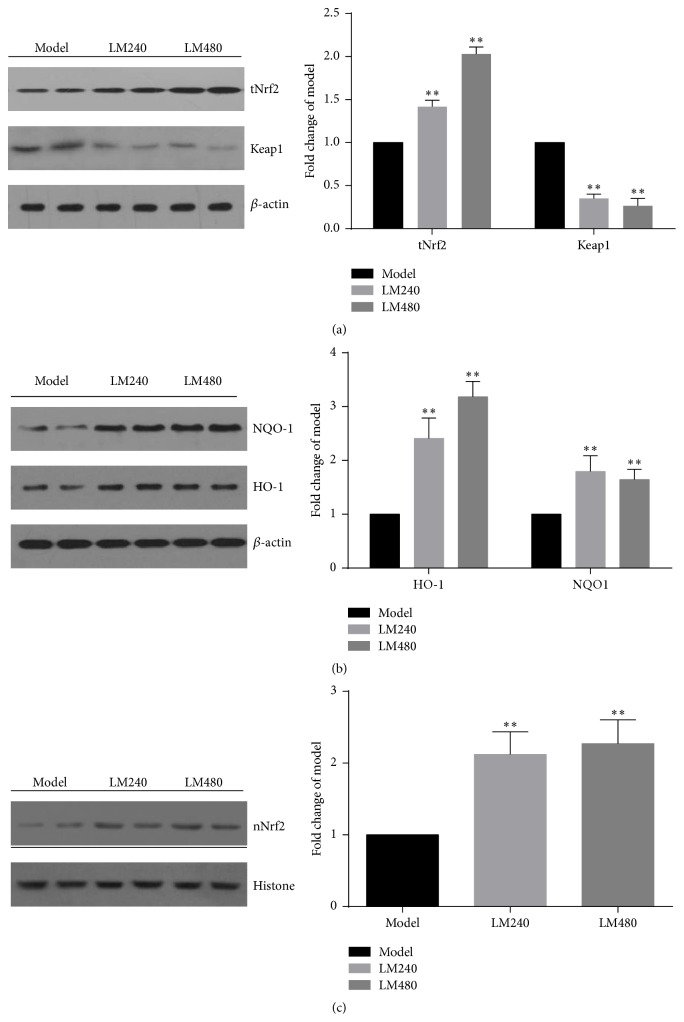
L-malate (LM) increases expression of total Nrf2 and nuclear Nrf2 protein and HO-1, NQO-1 protein after MIRI and decreases expression of Keap1 protein. Expression of total Nrf2, nuclear Nrf2, HO-1, NQO-1, and Keap1 was measured, with Western blot. Densitometric analysis was performed with Quantity One software 24 h later. Data are presented as means ± SD from 3 experiments. ^*∗∗*^ *P* < 0.01 versus model.

To better understand the mechanism of L-malate on cardioprotection, expressions of Keap1, Nrf2, HO-1, NQO-1, and Nrf2 in nuclear expression in ischemic myocardial tissues were measured using Western blot (Figure 6). Compared with model controls, Nrf2, HO-1, and NQO-1 expression were increased after the higher concentrations of L-malate (240 mg/kg: 1.42-fold, 1.80-fold, and 2.41-fold, *P* < 0.01; 480 mg/kg: 2.03-fold, 1.64-fold, and 3.18-fold, *P* < 0.01). Keap1 expression decreased after L-malate treatment at 240 mg/kg (0.35-fold, *P* < 0.01) and 480 mg/kg (0.27-fold, *P* < 0.01). Nrf2 in nuclear protein was significantly elevated after L-malate at 240 mg/kg (2.12-fold, *P* < 0.01) and 480 mg/kg (2.27-fold, *P* < 0.01), indicating increased Nrf2 nuclear translocation.

## 4. Discussion

When myocardial ischemia occurs, cardiac blood supply and attendant oxygen and nutrients are diminished [15]. Reestablishment of blood flow after prolonged ischemia can help to alleviate the initial injury but also it can aggravate myocardial damage and eventually cause structural and functional changes [16]. However, rational drug intervention prior to ischemia and reperfusion may reduce the degree of myocardial injury and promote recovery [17].

In the present study, the protective effect of L-malate on MIRI and its potential mechanism were revealed in rats. Data indicate that MIRI leads to necrosis and apoptosis in cardiomyocytes, which ultimately leads to myocardial infarction and cardiac loss of function. L-malate significantly reduced infract volume induced by MIRI and prevented depletion of cTn-I protein and LDH in ischemic heart tissues when administrated after MIRI. In addition, treatment with L-malate significantly preserved left ventricular function, as reflected by a significant increase in +*dp*/*dt*_max_, −*dp*/*dt*_max_, and LVSP and a decrease in LVEDP in the MIRI rat heart. These data clearly show that L-malate has a protective effect on MIRI, and this protective effect was dose-dependent up to a point.

MIRI is known to be caused by inflammation, oxidative stress injury, and apoptosis [2], and studies suggest that early inflammatory reactions and oxidative stress are two main pathological contributors to myocardial reperfusion injury [18, 19]. After inflammation is initiated, hs-CRP increases and inflammatory mediators exacerbate myocardial damage not only during acute ischemic injury, but also during the ensuing reperfusion phase [20–22]. TNF-*α*, IL-1, IL-6, and IL-8 after myocardial ischemia began to produce and release TNF*-α* [23, 24], which exacerbates myocardial injury via activation of neutrophils and endothelial cells [25]. Here, we observed that L-malate significantly decreased hs-CRP and TNF-*α*, compared to vehicle-treated animals (*P* < 0.05 or *P* < 0.01). Therefore, the protective effects of L-malate against MIRI may be due to suppression of the inflammatory response via inhibiting proinflammatory mediators. Oxidative stress injury through the generation of free radicals and reactive species can directly damage myocardial cells, resulting in cellular structural damage and cell death [26, 27]. Antioxidants are critical for response to endogenous or exogenous oxidative stress, and molecules such as GSH-PX and SOD can be synergistically protective [28, 29]. Therefore, increasing antioxidant activity may be helpful. L-malate significantly improved SOD and GSH-Px activity (*P* < 0.05 or *P* < 0.01) after MIRI suggesting that L-malate can reduce oxidative stress damage after MIRI.

As a transcription factor, Nrf2-mediated regulation of antioxidant and anti-inflammatory mediators is important for defense against oxidative stress [30]. Nrf2 is localized to the cytoplasm as an inactive complex bound to a repressor molecule, Keap1, which facilitates its ubiquitination, such that cytoplasmic Nrf2 is degraded by the ubiquitin pathway and cannot move to the nucleus [31]. Under oxidative or electrophilic stress, Nrf2 dissociates from the Keap1-Nrf2 complex, and when Nrf2 is not ubiquitinated, it can accumulate cytoplasmically and translocate to the nucleus. There, it can promote antioxidant genes such as NQO-1 and HO-1 and initiate their transcription [32, 33]. Our results indicate that L-malate significantly upregulated expression of total Nrf2, nuclear Nrf2, HO-1, and NQO-1, suggesting that L-malate could promote Nrf2 nuclear transfer, increase HO-1 and NQO-1, and reduce I/R injury. Keap1 is the protein primarily responsible for the regulation of Nrf2 by forming a homodimer responsible for sequestering Nrf2 in the cytosol and rendering it inactive [34]. The activity of Nrf2 is primarily regulated via its interaction with Keap1, which directs the transcription factor for proteasomal degradation. Keap1 expression was significantly downregulated by L-malate (240 and 480 mg/kg) relative to model controls, but Nrf2 can be regulated independently of Keap1 at the level of protein transcription [35, 36]; it can also be regulated at the level of translation and by posttranslational modifications, including phosphorylation of Nrf2 by interaction with epigenetic factors (micro-RNAs 144, 28 [37, 38]), various protein kinases (PKC, GSK-3b [39, 40]), and other protein partners (p21, caveolin-1 [41, 42]). These and other processes are potentially important determinants of Nrf2 activity with Keap1-independent pathway. So further research into L-malate as a preconditioning protectant of cardiovascular function is warranted.

In conclusion, L-malate can protect the heart against MIRI via anti-inflammatory and antioxidant activity and through Keap1/Nrf2-ARE pathway. Thus, L-malate may offer therapeutic efficacy for limiting the severity and functional deficits associated with MIRI.

## Figures and Tables

**Table 1:** Hemodynamic assessment under sham-operated conditions.

Groups	LVSP (mmHg)	LVEDP (mmHg)	+*dp*/*dt*_max⁡_ (mmHg/s)	−*dp*/*dt*_max _ (mmHg/s)
Sham	112.25 ± 5.06	10.88 ± 1.13	4532.13 ± 177.30	−3395.13 ± 254.31
Model	113.50 ± 6.74	10.75 ± 1.04	4592.63 ± 195.67	−3431.63 ± 258.17
DMF	116.00 ± 6.78	11.00 ± 1.31	4512.75 ± 159.85	−3573.50 ± 226.15
LM (15 mg/kg)	116.50 ± 6.14	10.88 ± 1.46	4575.88 ± 190.90	−3464.88 ± 242.18
LM (60 mg/kg)	118 ± 5.29	11.00 ± 1.00	4648.67 ± 209.13	−3446.33 ± 209.13
LM (120 mg/kg)	114.13 ± 7.74	11.00 ± 1.85	4605.38 ± 220.12	−3569.50 ± 274.68
LM (240 mg/kg)	115.30 ± 4.25	11.40 ± 1.27	4659.25 ± 200.57	−3606.21 ± 211.73
LM (480 mg/kg)	116.38 ± 9.78	11.00 ± 1.31	4550.75 ± 199.46	−3526.13 ± 234.33

Data are presented as the means ± SD. No significant differences were detected among groups. LVSP: left ventricular systolic pressure; LVEDP: left ventricular end-diastolic pressure; +*dp*/*dt*_max⁡_: indices of left ventricular contraction; −*dp*/*dt*_max⁡_: indices of left ventricular relaxation.
